# Lung carcinoma–associated cognitive impairment in a patient with Alzheimer’s disease pathology: A case report

**DOI:** 10.1002/ccr3.4482

**Published:** 2021-07-06

**Authors:** Kiyoto Takigawa, Masahiko Takaya, Kazunari Ishii, Kazumasa Saigoh, Osamu Shirakawa

**Affiliations:** ^1^ Department of Neuropsychiatry Faculty of Medicine Kindai University Osaka Japan; ^2^ Department of Radiology Faculty of Medicine Kindai University Osaka Japan; ^3^ Department of Neurology Faculty of Medicine and Department of Clinical Genetics Faculty of Medicine Kindai University Osaka Japan

**Keywords:** ^11^C‐Pittsburgh compound‐B, ^18^F‐THK5351, adenosquamous, Alzheimer's disease, cognitive impairment, lung carcinoma, positron emission tomography

## Abstract

A patient with Alzheimer's disease (AD) pathology when cognitive impairment is detected tends to be diagnosed with AD. However, before diagnosing, we make an effort to exclude other diseases, for example, carcinoma.

## Graphical abstract

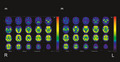

## 1 INTRODUCTION AND BACKGROUND

The pathological amyloid‐β and tau protein accumulation in the cerebral cortex does not necessarily lead to Alzheimer's disease (AD) diagnosis. Our current report presents a patient with an AD pathology showing general cognitive impairment considered to be derived from the lung carcinoma without metastasis.

Alzheimer's disease (AD), the most common neurodegenerative disease accompanying dementia, is considered to be caused by the deposition of pathological amyloid‐β and the successive pathological tau protein. The main clinical features of AD are an amnestic presentation, including learning impairment and problem recalling recently learned information, and nonamnestic impairments.^1^ Magnetic resonance (MR) imaging showed regional atrophy in the medial temporal lobes, and [^123^I] iodoamphetamine single‐photon emission computed tomography (IMP‐SPECT) showed hypoperfusion in the parietotemporal cortices and posterior cingulate gyrus/precuneus. Mild cognitive impairment (MCI), the prodromal stage of dementia, with amnestic MCI is considered to be due to AD, as supported by pathophysiological evidence.^2^

Sleep apnea syndrome (SAS) can cause cognitive impairment, although continuous positive airway pressure (CPAP) treatment could improve it.^3^ Additionally, cancer in noncentral nervous system malignancies can cause cancer‐related cognitive impairment.^4^

We present a case of a 64‐year‐old right‐handed Japanese man suspected of early‐stage MCI due to AD based on the pathophysiological findings. He also suffered from SAS and lung carcinoma without metastasis. Despite successful SAS treatment, his cognitive impairment was getting worse. After the surgical removal of the tumor on his right lung, his cognitive impairment improved. Now, we present the case with important implications.

## 2 CASE PRESENTATION

**FIGURE 1:**
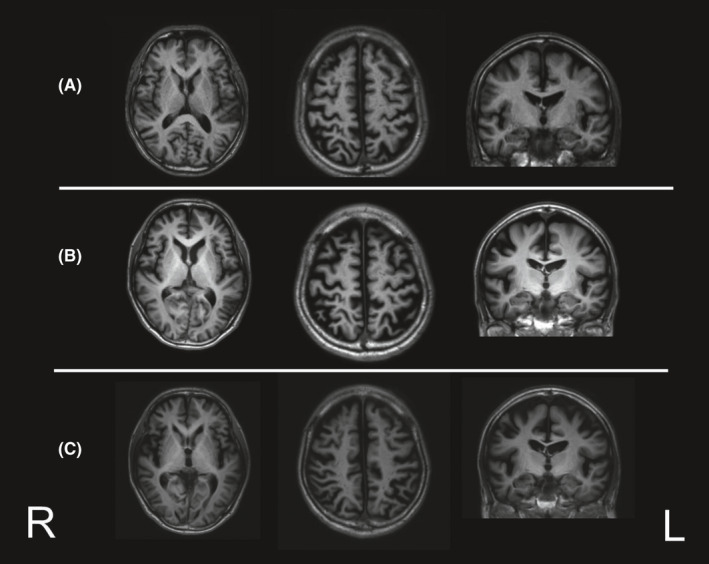
Magnetic resonance images (A) at baseline, (B) 18 months later, and (C) 3 years later. The bilateral superior parietal lobes show slight atrophy for his age, suggesting Alzheimer's disease pattern

**FIGURE 2:**
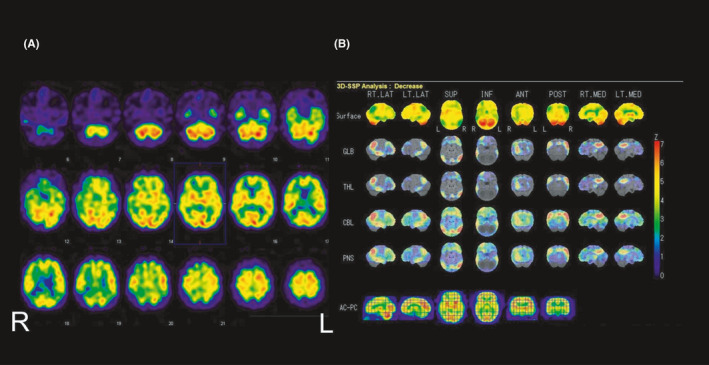
[^123^I] iodoamphetamine single‐photon emission computed tomography images at baseline; (A) axial images at baseline and (B) voxel‐based analysis (using the 3D SSP software) of the images. Both (A) and (B) show a hypoperfusion pattern of Alzheimer's disease, especially in the bilateral parietotemporal association and frontal association areas, and posterior cingulate gyrus and precuneus

The wife of a 64‐year‐old right‐handed Japanese man complained of the deteriorating cognitive function of his husband. For example, he had difficulty performing simple calculations and could not recognize his old friends. His medical history showed no illnesses, and he had no previous history of psychiatric disorders. He was a high school graduate. His wife considered that these impairments could be due to the problems at his job. He had begun talking about eagerly quitting his job and complaining about not feeling well. His wife suspected memory impairment in his daily life. When he visited our clinic with his wife, the patient calmly expressed not being well‐accustomed to his new job. His mental condition showed nothing in particular, but his episodic memory was found to be slightly impaired based on the medical interviews by a certified psychiatrist. Neurocognitive tests, MR imaging, and IMP‐SPECT were therefore performed (Table 1, Figures 1A and 2A,B). The neurocognitive test results showed a normal cognitive ability (Table 1). But the structural and functional brain images suggested an AD pattern (Figures 1A and 2A,B). It was because the bilateral superior parietal lobes showed slight atrophy for his age on MR images and hypoperfusion was observed in the bilateral parietotemporal association and frontal association areas and the posterior cingulate gyrus and precuneus, predominantly in the right cerebral hemisphere, based on IMP‐SPECT (Figures 1A and 2A,B).

**TABLE 1:** Neuropsychological evaluations

	Baseline	1 year later	18 months later	2 years later	3 years later
MMSE	28/30	24/30	23/30	27/30	25/30
Delayed recall	3/3	3/3	2/3	3/3	3/3
ADAS‐cog	6/70	15/70	6.6/70	8.4/70	13.7/70
CDT	N/A	13/15	N/A	14/15	13/15
WMS‐R					
Logical memory Ⅰ	N/A	15/50	18/50	19/50	16/50
Logical memory Ⅱ	N/A	11/50	12/50	12/50	12/50
FAB	N/A	8/18	15/18	13/18	12/18
WAIS‐Ⅲ					
Digit span forward	N/A	9/16	N/A	9/16	10/16
Digit span backward	N/A	5/14	N/A	4/14	5/14
ROCFT					
Copy	N/A	36/36	N/A	36/36	34/36
Delayed recall	N/A	15/36	N/A	10/36	11/36
JART	N/A	41/50	N/A	40/50	37/50

Abbreviations: ADAS‐cog, cognitive subscale of the Alzheimer's Disease Assessment Scale Japanese version; CDT, clock‐drawing test; FAB, frontal assessment battery; JART, Japanese Adult Reading Test; MMSE, Mini‐Mental State Examination; ROCFT, Rey‐Osterrieth Complex Figure Test; WAIS‐Ⅲ, Wechsler Adult Intelligence Scale, third edition; WMS‐R, Wechsler Memory Scale‐revised.

Three months after the first visit, the attending psychiatrist suspected that the patient might have SAS, based on the medical interviews. The psychiatrist referred the patient to a medical sleep center, where the patient was diagnosed with moderate to almost severe SAS based on the results of a sleep polysomnography test, which were as follows: apnea‐hypopnea index, 29.3; oxygen desaturation index, 17.3; minimum oxygen saturation, 93%; and arousal index, 38.0. CPAP was therefore introduced, which improved his SAS.

**FIGURE 3:**
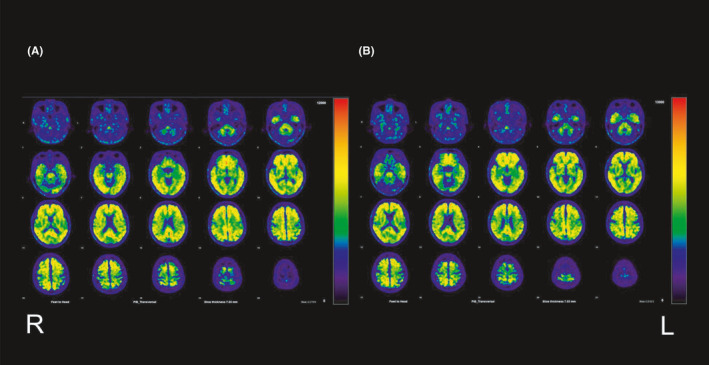
^11^C‐Pittsburgh compound‐B (PiB) positron emission tomography images: (A) 18 months and (B) 3 years later. Amyloid‐β accumulation is observed in the frontal, parietotemporal, and lateral occipital lobes, posterior cingulate and precuneus and striata. (A) and (B) images show a similar degree of pathological amyloid‐β accumulation

**FIGURE 4:**
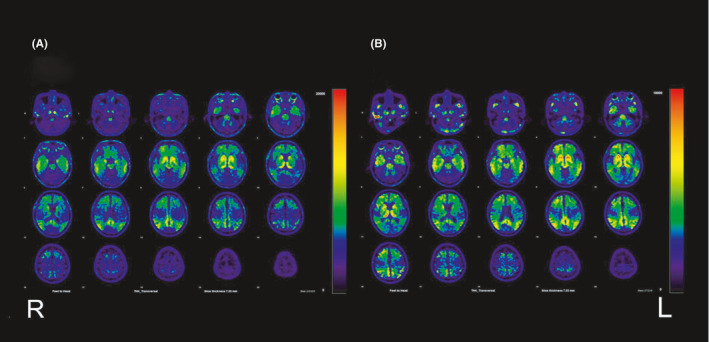
^18^F‐THK5351 positron emission tomography images: (A) 18 months and (B) 3 years later. THK5351 accumulation is shown in the bilateral medial temporal lobes, parietotemporal association and frontal association areas, and posterior cingulate gyrus and precuneus. The image in (B) shows a little progression of pathological THK5351 accumulation compared to those in (A)

The patient underwent neurocognitive tests at 1 year and at 18 months post‐baseline visit (Table 1), which showed, during the sixth month, gradually worsening scores on the Mini‐Mental State Examination (MMSE). He was diagnosed with nonamnestic MCI or early‐stage AD. Eighteen months post‐baseline visit, the patient also underwent ^11^C‐Pittsburgh compound‐B (PiB) positron emission tomography (PET) and ^18^F‐THK5351 (THK5351) PET, which revealed positive pathological amyloid‐β and tau protein accumulation (Figures 3A and 4A), thereby indicating AD neurological disease.^5^, ^6^ At that point, the results of MR imaging were almost identical to those of the baseline visit (Figure 1A,B).

Nineteen months after the baseline visit, lung carcinoma (right, S2, T3N0M0) was detected in the patient, but his only clinical feature was a dry cough, which his wife stated appeared occasionally. No metastasis was found, the tumor was surgically removed, and the pathology indicated adenosquamous carcinoma. Per the surgeon's instructions, the patient ceased the CPAP therapy for 2 months after the surgery.

Two years after the baseline visit, the patient's MMSE score showed that his general cognitive ability had improved after the resection surgery for his lung carcinoma (Table 1). However, his neurocognitive test scores were slightly lower 3 years post‐baseline visit than those 2 years post‐baseline visit (Table 1). The MR, PiB‐PET, and THK5351‐PET images are shown in Figures 1C, 3B and 4B, respectively. The results of MR imaging were almost identical to those performed at baseline visit and at 18 months post‐baseline visit (Figure 1A,B,C). However, the deposition of pathological tau protein had slightly increased. The time line is showed in Table 2.

**TABLE 2:** Timeline

1	2	3	4	5	6	7
Baseline visit	3 months from baseline visit	1 year from baseline visit	18 months from baseline visit	19 months from baseline visit	2 years from baseline visit	3 years from baseline visit
Performance of neuropsychological tests, brain MR imaging, and IMP‐SPECT	SAS diagnosis and start of CPAP	Performance of neuropsychological tests	Performance of neuropsychological tests, MR imaging, PiB‐PET, and THK5351‐PET	Lung carcinoma detection and surgical removal of the tumor. Interruption of CPAP for 2 months postoperative	Performance of neuropsychological tests	Performance of neuropsychological tests, MR imaging, PiB‐PET, and THK5351‐PET

Abbreviations: CPAP, continuous positive airway pressure; IMP‐SPECT, [^123^I] iodoamphetamine single‐photon emission computed tomography; MR, magnetic resonance; PET, positron emission tomography; PiB, ^11^C‐Pittsburgh compound‐B; SAS, sleep apnea syndrome; THK5351, ^18^F‐THK5351.

## 3 DISCUSSION

To our knowledge, our report is the first to show the finding in relation to lung carcinoma without metastasis and cognitive impairment. The noncentral nervous system cancer has been shown to induce cognitive impairment; however, its mechanism has not been well‐studied.^7^ Moreover, little is known about the cognitive function of patients with cancer prior to surgery. Only three studies have evaluated the cognitive function of the patients newly diagnosed with breast cancer.^7^

The patient's cognitive impairment was possibly caused by paraneoplastic syndrome associated with adenosquamous carcinoma.^8^ Other main possible causes for cognitive impairment in patients with carcinoma are metastasis to the brain, a side effect of chemotherapy, and comorbid depression. His lung carcinoma did not spread to his brain, as described earlier; he did not undergo chemotherapy, and he was not diagnosed with any mental disorders, according to the *Diagnostic and Statistical Manual of Mental Disorders: Fifth Edition*. Therefore, we cannot help considering his cognitive impairment might be caused by carcinoma itself. However, the detailed mechanism of cognitive impairment in our case remains to be investigated in the future.

The SAS and pathophysiological evidence in isolation cannot fully explain the patient's cognitive impairment. SAS, especially its obstructive form, can induce cognitive impairment, in association with general cognitive impairment.^9^ Our current case presented general cognitive impairment 1 year post‐baseline visit, which gradually worsened, despite successful CPAP introduction. The patient did not show the typical cognitive impairment of AD, although the pathophysiological findings using PiB‐PET, THK5351‐PET, and IMP‐SPECT suggested an AD‐type impairment.

Our current report's first limitation is the difficulty to make replication in resource‐poor settings. Our methods need such manpower skills, equipment, and facilities, which are not available in small‐scale hospitals. The second is only having one case, although we observed the patient longitudinally and in detail. Therefore, not concluding that lung cancer has caused the patient's cognitive impairment is better. Cohort studies investigating noncentral nervous system cancers, divided by type, remain to be performed.

## CONFLICTS OF INTERESTS

None declared.

## AUTHOR CONTRIBUTIONS

KT performed the literature search, created the tables and figures, and wrote the manuscript. MT conceived the idea, directed and planned the study, performed the literature search, analyzed the neurocognitive data, created the tables and figures, and revised and submitted the manuscript. KI managed the neuroimaging examinations and analyzed the neuroimages. KT, MT, and KS obtained the informed consent. OS provided useful comments for the study and manuscript.

## ETHICAL APPROVAL

Written informed consent was obtained from both the patient and the patient's spouse to publish this report.

## Data Availability

All available data are reported in this manuscript.
